# Inter-rater reliability of the Foot Posture Index (FPI-6) in the assessment of the paediatric foot

**DOI:** 10.1186/1757-1146-2-26

**Published:** 2009-10-21

**Authors:** Stewart C Morrison, Jill Ferrari

**Affiliations:** 1 The University of East London, Stratford Campus, Water Lane, London, E15 4LZ, UK

## Abstract

**Background:**

Reliability is an integral component of clinical assessment and necessary for establishing baseline data, monitoring treatment outcomes and providing robust research findings. In the podiatric literature traditional measures of foot assessment have been shown to be largely unreliable. The Foot Posture Index (FPI-6) is a clinical tool used in the assessment of foot and to date, there is limited research published which evaluates the reliability of this tool in children and adolescents.

**Method:**

Thirty participants aged 5 - 16 years were recruited for the research. Two raters independently recorded the FPI-6 score for each participant.

**Results:**

Almost perfect agreement between the two raters was identified following weighted kappa analysis (Kw = 0.86).

**Conclusion:**

The FPI-6 is a quick, simple and reliable clinical tool which has demonstrated excellent inter-rater reliability when used in the assessment of the paediatric foot.

## Background

The clinician has become increasingly aware of the need to have valid and reliable measures of assessing foot position for establishing baseline data, monitoring treatment outcomes and providing robust research findings. Most of the common measures of foot posture have been scrutinised for validity and reliability in the adult foot [1-4] but very little attention has been given to establishing the usefulness of similar measures in the assessment of the paediatric foot. While it is a broad assumption, clinicians often believe that the outcomes of validity and reliability studies of measures of foot position in adults are directly transferable to paediatric populations. This may not be the case and the extrapolation of such findings would be erroneous.

In an extensive study on the reliability of foot position in children (4-6 years), adolescents (8 - 15 years) and adults (20 - 50 years), it was reported that the reliability of measures of foot position in children were reduced when compared to adults, with raters commenting that children remained less still between repeated measures [5]. The reduced reliability of measures of foot position in children was also identified in a later study looking at the intra-rater reliability of measuring anthropometric characteristics of children's feet [6]. Since clinicians rarely need to take repeated measures at each assessment session but are more interested in the comparability of a measurement on a subsequent visit, future reliability studies may need to consider asking all participants to move about between repeated measures. It may be that children are generally less consistent in the placement of their feet compared to adults, thus reducing measurement reliability.

The Foot Posture Index (FPI-6) is an assessment tool that is thought to reduce many of the reliability concerns surrounding more traditional measures of the foot. The FPI-6 has been refined from an eight point scale to a six point scale and permits assessment across the three planes of the foot [4]. The FPI-6 yields a score based upon six individual criteria which are summated to provide a total score which is then used to determine foot posture. This total score is often used in the form of continuous data, however this assumes that each individual item of the index and the divisions within that item have equal weighting. This is not based upon any evidence and it is believed that this has been formed for convenience.

The reliability of the FPI-6 has been tested in adults with excellent intra-rater results (ICC 0.92 - 0.93) but moderate inter-rater results (0.52 - 0.65) [7]. Two studies investigating the reliability of the index in a paediatric population have been identified, one of which evaluated the reliability of the older version of the index (FPI-8) [5]. This study looked at a number of measures of foot position in addition to the FPI-8 and following reliability analysis, ICC values of 0.80 for children and 0.91 for adolescents were presented. More recently, Cain et al [8] investigated the intra-rater and inter-rater reliability of the refined FPI-6 on ten adolescents. Findings from this study reported excellent intra-rater reliability (ICC values ranged from 0.81 - 0.92) and good inter-rater reliability (ICC 0.69). However, consideration of the nature of the data generated by the FPI-6 would suggest that analysis using ICCs would be incorrect for the present study unless logit transformed scores are used. This is the process of changing raw FPI-6 scores into a data form suitable for parametric analysis but for this, large data sets are required [9]. Without transformation the index produces categorical data and therefore raw scores should be analysed using Kappa scores, particularly when the data is not normally distributed [10].

In clinical practice it is common that patient care is shared amongst a team of clinicians and therefore, it is vital that any tool used in the assessment of the child is repeatable between clinicians. There is limited evidence looking at the reliability of traditional measures of foot posture in children, however initial research suggests that the FPI-6 is a reliable tool when used in the assessment of the child's foot. This study aims to investigate the inter-rater reliability of the FPI-6 when used by two experienced observers in the assessment of the paediatric foot.

## Methods

### Participants

A convenience sample of 30 participants aged 5 to 16 years of age was recruited for the study from paediatric clinics at the Clinical Education Centre for Podiatric Medicine at the University of East London, UK. Prior to data collection ethical approval was granted from the University of East London. Details of the research were sent to parents/guardians with the appointment information and on attendance, parents/guardians gave informed consent form for participation. The children also assented to participate in the study.

All children referred to the paediatric clinic were considered for inclusion. Children were excluded if they presented with a foot position that would be associated with abnormal structural features or would obscure visualisation of normal foot architecture (for example, congenital foot deformity such as talipes, history of surgery, Juvenile Idiopathic Arthritis).

### Procedure

Inter-rater reliability was determined for two podiatrists with postgraduate experience of working in paediatrics (in excess of five years). Both raters participated in a training session on the FPI-6 and had equal exposure to the index prior to start of the study. The training session was undertaken on two participants (not included in the study) for familiarisation with the assessment tool and to allow open discussion about the index criteria.

On the day of attendance for podiatric assessment, consent and assent was determined prior to starting data collection. Each participant was asked to stand, take a few steps forward and march on the spot for six-eight steps and then to stand still, with arms by their side and looking forward. Both observers performed an independent bilateral foot assessment of each child using the six criteria of the FPI-6:

▫ talar head palpation

▫ curvature at the lateral malleoli

▫ inversion/eversion of the calcaneus

▫ talonavicular bulging

▫ congruence of the medial longitudinal arch

▫ abduction/adduction of the forefoot on the rearfoot

Since bias may be increased when measuring consecutively between the left and right feet, the first foot measured was always randomly chosen. The child remained in the same position whilst the second observer assessed foot posture. Each observer was blinded to the other observer's results.

### Data Analysis

Data were entered and analyses were performed using SPSS Software Package version 15.0 and MedCalc statistical software. Before conducting analysis, the data were tested for normality using the Kolmogorov-Smirnov test. The data were not normally distributed and were positively skewed. The FPI-6 values for the left and right foot for each rater were compared using a Wilcoxon matched paired signed ranks test. There was no significant difference between the left and right foot for rater one (z = -0.49, p = 0.62) or rater two (z = -0.22, p = 0.83). Therefore for further analysis, the left foot only was considered.

## Results

Thirty participants were recruited into the research and further information on gender and age-range is presented in Table 1. Scores of foot posture are presented in Table 2. These scores are presented for each participant and from both raters.

**Table 1:** Gender and age banding for participants recruited into the research (n = number of participants in age band)

**Age band (years)**	**n**	**Male**	**Female**
5-6	5	3	2
7-8	4	4	0
9-10	10	4	6
11-12	0	0	0
13-14	8	3	5
15-16	3	0	3
Total	30	14	16

**Table 2:** Raw FPI-6 scores for individual participants involved in the study from both raters.

	**FPI-6 Scores**	
**Participant**	**Rater 1**	**Rater 2**
1	11	8
2	11	12
3	8	8
4	11	12
5	10	10
6	5	3
7	6	6
8	11	11
9	8	7
10	11	11
11	12	12
12	10	10
13	5	5
14	12	12
15	9	10
16	7	8
17	11	11
18	10	10
19	10	10
20	7	7
21	1	2
22	5	4
23	9	10
24	-1	-1
25	12	12
26	11	11
27	7	6
28	5	5
29	9	9
30	11	11

The FPI-6 score was assigned to a predetermined category (highly pronated (FPI-6 score 10 to 12), pronated (FPI-6 score 6 to 9), neutral (FPI-6 score 0 to 5), supinated (FPI-6 score -1 to -4) and highly supinated (FPI-6 score -5 to -12) as recommended by Redmond [11]. Table 3 shows the observed agreement between the two raters for each foot type category. A weighted kappa score was applied to the actual scores and a Kappa coefficient (Kw) of 0.86 was determined. The result has been described as almost perfect agreement [12,13]. Agreement between raters for this categorical data into foot type categories was also tested using a weighted kappa test. The inter-rater reliability showed almost perfect agreement (Kw = 0.88).

**Table 3:** Level of agreement in determining foot posture between the two raters

		**Observer A**			
**Observer B**	**HP**	**P**	**N**	**S**	**Total**
HP	14	2	0	0	16
P	1	7	0	0	8
N	0	0	5	0	5
S	0	0	0	1	1
Total	15	9	5	1	30
				**Weighted Kappa**	0.88

HP = Highly Pronated (10 - 12)

P = Pronated (6 -9)

N = Neutral (0 - 5)

S = Supinated (-1 - -4)

## Discussion

The aim of this study was to determine the inter-rater reliability of the FPI-6 in the assessment of the paediatric foot. The inter-rater agreement when the actual score was compared and when the score was categorised showed almost perfect agreement [13]. One previous study looking at the reliability of the FPI-6 in adolescents also determined good level of inter-rater reliability (ICC = 0.69) [8]. This study looked at intra and inter-rater reliability across three raters, however direct comparison of results is difficult because in this study the inter-rater reliability was measured using an Interclass Correlation Coefficient (ICC).

Using the FPI-6 in adults, Cornwall *et al *reported moderate reliability between observers [7]. The study had a substantial sample size (n = 46) and also used three raters (of varying clinical experience) to determine inter-rater reliability, left and right foot data was pooled and an ICC applied. The study reported moderate agreement between observers (ICC = 0.57) for actual FPI-6 scores and between 65-74% agreement when the FPI-6 scores were categorised. The findings reported must be interpreted with caution following the pooling of data as this is a procedure that has been considered to give false results [14].

It is likely that the sample size of the present study and the experience of the raters using the index accounts for the high level of inter-rater agreement. Cornwall *et al *reported that a learning effect was seen whereby the ICC improved for the second half of the measurements compared to the first [7]. In the present study, both raters had similar experience with using the FPI-6 which is important because otherwise, varying levels of expertise would render the kappa an inappropriate tool for analysis [15]. As the raters both had experience in the paediatric field of podiatry they were able to develop a good rapport and allow the children to relax whilst data collection occurred. Care was taken to ensure that the children remained still between repeated observations, a problem noted in one of the earlier studies [5].

An improvement in inter-rater reliability would be expected to be seen when using the categorical ratings as recommended in the user manual as this introduces a smoothing effect of the differences between observers. This is due to each category covering a range of two to four points which will be within the disagreement margin for the actual values between raters. The categorical ratings have recently been updated [16] but the new groupings do not allow for differences between the potentially abnormal and pathological scores (previously called pronated and highly pronated) and therefore were not used in this study.

A further consideration for the differing results between the studies is the foot type assessed. The adult FPI-6 study [7] included the greatest range of foot types with FPI-6 scores within the categories of "supinated" through to "pronated" and included one "highly pronated" case. The authors commented that all raters had difficultly distinguishing in the mid-range of the index - between normal/pronated feet and normal/supinated feet - which was where the majority of their participant group were placed. The FPI-8 study in children [5] had values ranging from -1 to +14 thus including only "normal" feet through to "highly pronated" feet and so had less need to differentiate in the mid-range of the index. The present study, because of its selection of a convenience sample of children attending for podiatric treatment, was only able to assess the reliability in the end range of the scale, including no children with highly supinated feet. Having only participants in this small range may have increased the inter-rater reliability and it is recognised that the reliability does need to be tested across the full range of the index. However, in this study the score was tested for the typical group seen for treatment and research purposes.

A limitation to this study was the sample size. A sample size of 30 cases with two raters is an acceptable minimum sample size for when a moderate level or higher kappa coefficient is expected [15], and to show that kappa is different from a value of zero. To confirm the inter-reliability of the FPI-6 in children, further data should be collected, using a larger range of foot types and also testing in specific groups in which treatment or research is occurring such as in cerebral palsy or hypermobility syndromes. Intra-rater reliability must also be considered.

## Conclusion

The findings of this study show the FPI-6 has almost perfect inter-rater reliability (Kw = 0.86) between two experienced practitioners when used on the paediatric foot. This suggests that the FPI-6 may be of value in clinical practice and for use in podiatric research. Good inter-rater reliability provides confidence in this assessment tool; however reliability isn't solely a measure of the instrument. One must take into consideration the instrument, the practitioner, the situation and the participant. To ensure good inter-rater reliability for paediatric participants, all raters must receive similar training and have experience in treating the paediatric patient so the recording of outcome measurements is optimised.

## Competing interests

The authors declare that they have no competing interests.

## Authors' contributions

SM and JF were responsible for the conception and design the project. They have collected the data, conducted the statistical analysis and written the manuscript. Both authors have read and approved the final manuscript.
